# The landscape of human p53‐regulated long non‐coding RNAs reveals critical host gene co‐regulation

**DOI:** 10.1002/1878-0261.13405

**Published:** 2023-03-09

**Authors:** Martin Fischer, Konstantin Riege, Steve Hoffmann

**Affiliations:** ^1^ Computational Biology Group Leibniz Institute on Aging – Fritz Lipmann Institute (FLI) Jena Germany

**Keywords:** DREAM complex, lncRNA, nested genes, p53, resource, retinoblastoma protein RB

## Abstract

The role of long non‐coding RNAs (lncRNAs) in p53‐mediated tumor suppression has become increasingly appreciated in the past decade. Thus, the identification of p53‐regulated lncRNAs can be a promising starting point to select and prioritize lncRNAs for functional analyses. By integrating transcriptome and transcription factor‐binding data, we identified 379 lncRNAs that are recurrently differentially regulated by p53. Dissecting the mechanisms by which p53 regulates many of them, we identified sets of lncRNAs regulated either directly by p53 or indirectly through the p53‐RFX7 and p53‐p21‐DREAM/RB:E2F pathways. Importantly, we identified multiple p53‐responsive lncRNAs that are co‐regulated with their protein‐coding host genes, revealing an important mechanism by which p53 may regulate lncRNAs. Further analysis of transcriptome data and clinical data from cancer patients showed that recurrently p53‐regulated lncRNAs are associated with patient survival. Together, the integrative analysis of the landscape of p53‐regulated lncRNAs provides a powerful resource facilitating the identification of lncRNA function and displays the mechanisms of p53‐dependent regulation that could be exploited for developing anticancer approaches.

## Graphical abstract

By integrating transcriptome and transcription factor‐binding data, we identified long non‐coding RNAs (lncRNAs) that are recurrently differentially regulated by p53. We identified sets of lncRNAs regulated either directly by p53 or indirectly through the p53‐RFX7 and p53‐p21‐DREAM/RB:E2F pathways. Moreover, we identified lncRNAs that are regulated through p53‐bound enhancers or co‐regulated with their protein‐coding host genes.

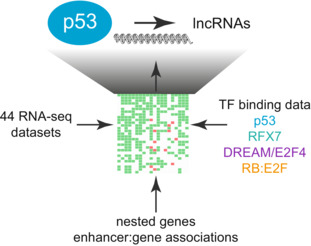

**Abbreviations**
DREAM: DP, RB‐like, E2F4, and MuvB
HFF: human foreskin fibroblasts
lncRNA: long non‐coding RNA
ncRNA: non‐coding RNA
p21: cyclin‐dependent kinase (CDK) inhibitor p21
p53: tumor suppressor protein p53
PH: proportional hazards
RB: retinoblastoma protein
RFX7: regulatory factor X 7
snoRNA: small nucleolar RNA
TCGA: The Cancer Genome Atlas
TSS: transcription start site

## 1 Introduction

The transcription factor p53 primarily exerts its function as a tumor suppressor by regulating the expression of other genes [1, 2, 3]. Although p53 is arguably one of the best‐studied proteins, it remains unclear how p53 regulates many of its targets [3]. We started to disentangle the p53 gene regulatory network into subnetworks of genes controlled either directly by p53 or indirectly through downstream transcription factors [4, 5]. Gene regulatory pathways utilized by p53 for indirect regulation include, for instance, the p53‐p21‐DREAM/RB:E2F pathway through which p53 down‐regulates cell cycle genes [4, 5, 6, 7, 8]. Via the p53‐RFX7 signaling axis, p53 induces a subnetwork of tumor suppressor genes [9]. While transcriptional regulation by p53 has been extensively studied for protein‐coding genes [1, 2, 3], there is a paucity of information on how p53 may regulate non‐coding RNAs (ncRNAs). The recent expansion of high‐throughput sequencing‐derived transcriptomic and epigenomic data substantially facilitates the investigation of this critical connection [10, 11, 12].

The human genome contains thousands of loci that can be transcribed into long non‐coding RNAs (lncRNAs), which are ncRNAs with a length > 200 nucleotides [13]. To date, many lncRNAs have been reported to be recurrently dysregulated or mutated in multiple pathological conditions including cancer [11, 12, 14, 15, 16]. So far, only a few lncRNAs have been characterized functionally as discerning functional lncRNAs from the vast number of transcribed ones remains a major challenge [17]. Importantly, lncRNAs have been found to contribute to essentially all hallmarks of cancer [18, 19]. They have been shown to have important roles in mediating p53's tumor suppressor function [18, 20, 21], *e.g.*, through the regulation of growth restriction, DNA repair, and apoptosis downstream of p53 [22, 23, 24, 25, 26, 27, 28, 29, 30]. Given that p53‐dependent regulation can be used to select and prioritize lncRNAs for functional analyses, multiple studies aimed at the identification of p53‐regulated lncRNAs across the genome in specific cell types [31, 32, 33, 34, 35, 36]. However, compared with protein‐coding genes, the expression of lncRNAs is highly tissue and cell‐type‐specific [10, 37, 38], calling for approaches that integrate transcriptome information from multiple sources. Recently, p53‐dependent gene regulation was investigated across tumors from mouse models, and lncRNAs recurrently regulated by p53 were found to have an important role in modulating the outcome downstream of p53 [39], but the findings cannot be readily translated to humans because the p53 gene regulatory network differs strongly between mice and humans [40, 41] and most lncRNAs are not evolutionarily conserved [37, 38, 42]. In the past, we employed integrative analyses of gene expression and transcription factor‐binding data and showed that a recurrent regulation of genes across cell types and conditions can be particularly powerful in identifying important nodes in gene regulatory networks [4, 40, 43, 44].

Here, we resorted to a collection of 44 RNA‐seq datasets on p53‐responsive gene regulation from human cell systems, to analyze the landscape of p53‐regulated lncRNAs across various cell types and treatment conditions. Our integrated dataset covers 9549 lncRNAs with expression data from multiple individual RNA‐seq datasets and reveals lncRNAs that are recurrently regulated by p53. Additionally, we integrated binding data (ChIP‐seq) on transcription factors known to have important roles in the gene regulatory network of p53.

## 2 Materials and methods

### 2.1 RNA‐seq datasets

RNA‐seq datasets were taken from our previously published collection of high‐throughput datasets on p53‐regulated transcriptomes [45]. The collection included 44 RNA‐seq datasets with quality control for p53 responsiveness. The reference genome was hg38 with its gene annotation from Ensembl v102 (GENCODE v36) [46]. RNA‐seq datasets from parental HCT116 (GSE162160 [9]), HCT116 p21^−/−^ (GSE77841 [4]), and human foreskin fibroblast (HFF) cells depleted of RB, p130, and p107 (GSE128711 and GSE135842 [8]) have been assessed in detail.

### 2.2 p53 Expression Score (RNA‐seq)

Following our meta‐analysis approach [4], a p53 Expression Score (RNA‐seq) was calculated for a gene as the number of RNA‐seq datasets that find it significantly up‐regulated minus the number of datasets that find the gene to be significantly down‐regulated by p53. To calculate the score, we required a gene to be sufficiently expressed in at least three datasets. A gene was deemed to be expressed when deseq2 was able to include it in the differential expression analysis, *i.e.*, assign log_2_fold‐change and FDR values, as described previously [45].

### 2.3 ChIP‐seq data integration

The p53 ChIP‐seq collection of 28 datasets has been published previously [40, 43]. Similarly, p53REs were taken from our previous study [43]. RFX7 ChIP‐seq data was taken from GSE162184 [9]. We included published collections of nine ChIP‐seq datasets on E2F4, four ChIP‐seq datasets on p130/p107, and six ChIP‐seq datasets on RB [45]. Genomic loci and transcription factor‐binding data were visualized using the UCSC Genome Browser [47] with track hubs provided through www.TargetGeneReg.org [45].

To identify genes recurrently bound by p53 near their transcription start site (TSS), we used the 7705 recurrent p53‐binding sites that previously were identified in at least 5 out of 28 p53 ChIP‐seq datasets [43] and we reported genes that harbor at least one such p53‐binding site within 2.5 kb from one of their known TSSs, a distance which has been established previously to be well‐suited to identify direct p53 target genes [4, 48]. To identify genes potentially regulated through p53‐bound enhancers, we overlapped our core set of 7705 recurrent p53‐binding sites with double‐elite enhancers listed in the GeneHancer collection [49]. Subsequently, we extracted genes with a double‐elite association to a p53‐bound enhancer that does not display p53‐binding within 2.5 kb from their TSS. E2F4, p130/p107, and RB ChIP‐seq datasets were joined using bedtools multiinter v2.30.0 [50]. A binding site was considered to be recurrent when it was identified in at least four out of nine, two out of four, and three out of six ChIP‐seq datasets on E2F4, p130/p107, and RB, respectively. To identify genes recurrently bound by the DREAM complex, we resorted to ChIP‐seq data on its key repressive components E2F4 and p130/p107. The overlap of recurrent binding sites of E2F4 and p130/p107 was identified using bedtools intersect and considered recurrent DREAM‐binding sites. Genes were reported that displayed recurrent binding sites of DREAM and RB within 1 kb of their TSS, a distance threshold established previously [4].

### 2.4 Host gene identification

LncRNAs nested within protein‐coding genes were inferred utilizing strand‐aware bedtools intersect v2.30.0 [50]. An overlap fraction of 1 was required for protein‐coding (biotype) genes from the Ensembl hg38 genome annotation v102 and the 9549 lncRNAs observed in our analysis.

### 2.5 Survival analysis

Survival analyses for The Cancer Genome Atlas (TCGA) cases were based on the expression of a set of 313 and 66 lncRNAs recurrently up and down‐regulated by p53, respectively. We retrieved clinical data and TPM normalized gene expression values for tumor samples, which could be unambiguously linked to somatic nucleotide variant annotation data, from TCGA v36.0 using the r package tcgabiolinks v2.25.0 [51]. Per patient, the p53 mutation state was assigned. For the whole pan‐cancer set comprising 33 cancer types, we calculated per sample expression scores for the lncRNA gene sets using the official genepattern codebase v10.0.3 for single‐sample gene set enrichment analysis (https://github.com/GSEA‐MSigDB/ssGSEA‐gpmodule) [52]. A single‐sample expression score measures the degree of coordinated up or down‐regulation of genes in a given set. Subsequently, we subdivided the expression scores into three equally sized categorial groups (high, medium, and low). Kaplan–Meier plots and multivariate Cox regression analysis based on the expression groups and controlled for confounders' age and sex, were performed on clinical time to event and event occurrence information using the r survival package v3.2‐13. The Cox proportional hazards (PH) model was used to investigate the relation between patient survival and categorical expression levels. The rates of occurrence of events over time were compared between the groups using the fitted PH model.

### 2.6 Statistics

Correlation of log_2_fold‐change values was assessed using the Spearman correlation. TPM values from RNA‐seq datasets were compared using a two‐sided unpaired Student's *t*‐test. Bar graphs display mean and standard deviation. *, **, ***, and n.s. indicate *P* values < 0.05, < 0.01, < 0.001, and > 0.05, respectively.

## 3 Results

### 3.1 Hundreds of lncRNAs are recurrently regulated by p53

**Fig. 1:**
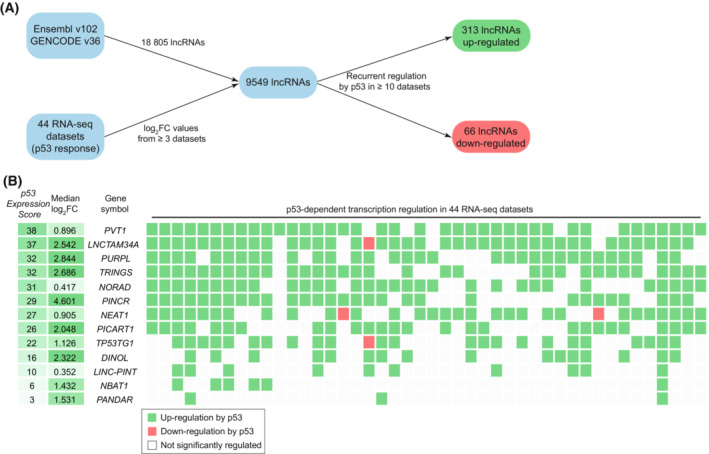
Identification of p53‐dependent lncRNA regulation. (A) Flow chart for the integration of 44 RNA‐seq datasets on p53‐dependent lncRNA regulation. Out of 18 805 lncRNAs listed in Ensembl v102/GENCODE v36, log_2_fold‐change (log_2_FC) values from at least three datasets were obtained for 9549 lncRNAs. Recurrent p53‐dependent regulation was specified by a p53 Expression Score (the number datasets with significant up‐regulation minus the number of datasets displaying significant down‐regulation by p53) ≥ 10 and ≤ −10 for up and down‐regulated lncRNAs, respectively. (B) The p53 Expression Score (RNA‐seq) and data from the underlying 44 individual RNA‐seq datasets, including the median log_2_fold‐change (log_2_FC; regardless of statistical significance), visualized for 13 selected lncRNAs known to be direct p53 targets. Green and red datapoints indicate significant up and down‐regulation, respectively, while white datapoints indicate no significant regulation by p53.

To comprehensively assess recurrent p53‐dependent lncRNA regulation, we performed a meta‐analysis of 44 RNA‐seq datasets derived from experiments that affected p53 signaling [45] (see Section 2). For each gene, we calculated a p53 Expression Score (RNA‐seq) based on these 44 datasets for each gene by the number of RNA‐seq datasets yielding significant up‐regulation minus the number of datasets with significant down‐regulation of the gene following p53 signaling (Table S1). The integrated dataset covers 9549 lncRNAs for which expression was observed in at least 3 out of 44 datasets (see Section 2, Fig. 1A). The results for 13 selected lncRNAs previously reported as p53 targets, namely *PVT1* [53], *LNCTAM34A* (also known as *GUARDIN*) [29], *PURPL* (*LINC01021*) [36, 54], *TRINGS* (*AC093866.1*) [55], *NORAD* [25], *PINCR* [56], *NEAT1* [26, 27], *PICART1* [57], *TP53TG1* [58], *DINOL* [24], *LINC‐PINT* [23], *NBAT1* [59], and *PANDAR* [22] (Fig. 1B), indicate that our integrated dataset recovers well *bona fide* p53‐regulated lncRNAs.

Notably, many lncRNAs do not reach significance thresholds in the individual datasets because of their very low abundance. We are addressing this limitation by directly assessing the lncRNA's median fold‐change, *i.e.*, the median of all fold‐change values. When the median fold‐change deviates substantially from zero, it can indicate recurrent up or down‐regulation of a gene although most individual data points did not reach the significance threshold. For instance, genes like the p53 target *PANDAR* show a high median fold‐change, indicating a recurrent up‐regulation despite only a few values reaching statistical significance (Fig. 1B).

To identify lncRNAs that are recurrently regulated by p53, we employed conservative p53 Expression Score thresholds of ≥ 10 and ≤ −10 for up and down‐regulated lncRNAs, respectively. These thresholds led to the identification of 313 and 66 lncRNAs that are recurrently up and down‐regulated by p53, respectively. The full dataset is available through Table S1. The top up‐ and down‐regulated lncRNAs are displayed in Fig. S1.

### 3.2 Mechanisms of lncRNA regulation by p53

#### 3.2.1 Identification of 86 lncRNAs directly targeted by p53

**Fig. 2:**
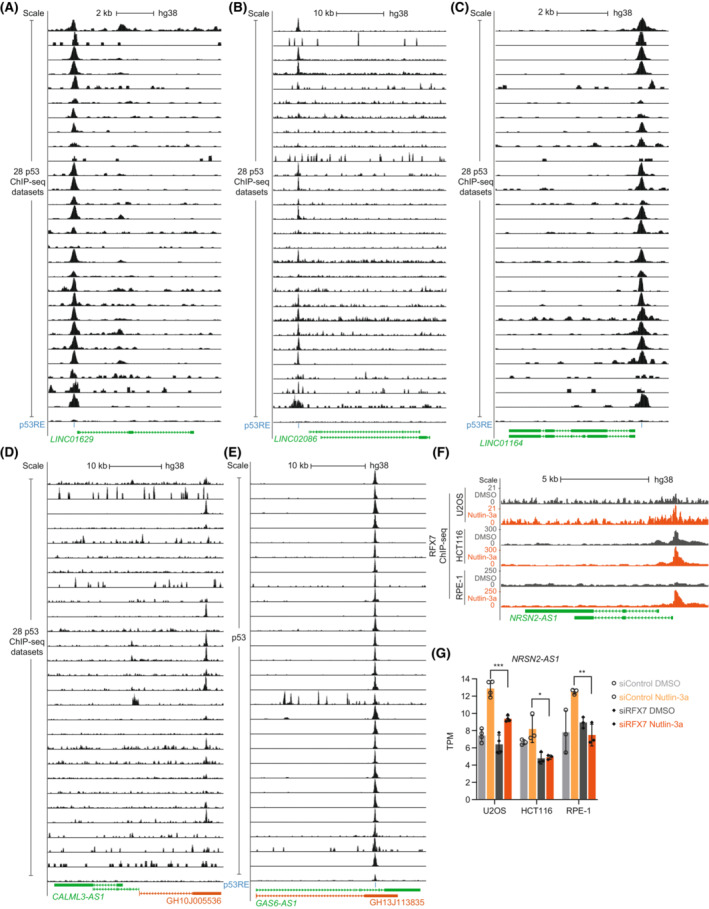
LncRNA regulation directly by p53 and indirectly through the p53‐RFX7 axis. Data on p53 binding from 28 ChIP‐seq datasets [40, 43, 45] and p53REs underlying the p53‐binding sites [43] at the genomic loci of the recurrently up‐regulated lncRNAs (A) *LINC01629*, (B) *LINC02086*, (C) *LINC01164*, (D) *CALML3‐AS1*, and (E) *GAS6‐AS1*. (D, E) Orange track contains enhancers from the GeneHancer double‐elite collection [49] and their gene association. (F) RFX7‐binding data derived from ChIP‐seq [9] at the *NRSN2‐AS1* locus. Gray and orange tracks show data from DMSO control and Nutlin‐3a‐treated cells, respectively. (G) Transcripts per million (TPM) values of *NRSN2‐AS1* obtained from RNA‐seq data of Nutlin‐3a (orange) and DMSO control‐treated (gray) cells transfected with siControl (low saturation) and siRFX7 (high saturation) [9]. Bar graphs display mean and standard deviation. TPM values from RNA‐seq datasets were compared using a two‐sided unpaired Student's *t*‐test. *, **, ***, and n.s. indicate *P* values < 0.05, < 0.01, < 0.001, and > 0.05, respectively.

The tumor suppressor p53 employs multiple mechanisms to regulate gene expression [1, 2, 3]. As a transcription factor, p53 can up‐regulate the expression of many target genes directly. This process typically involves p53 binding within a 2.5 kb region from their TSS [4, 48]. To identify lncRNAs under the direct control of p53, we integrated a collection of 7705 recurrent p53‐binding sites that we identified earlier based on 28 p53 ChIP‐seq datasets generated from multiple human cell lines [40, 43]. Out of the 313 lncRNAs recurrently up‐regulated by p53, we identified 86 that display a recurrent p53‐binding site close to their TSS (Table 1). While this set contains several lncRNAs previously reported to be under p53 control, the majority has not yet been proposed to be directly targeted by p53. For instance, we propose that *LINC01629*, *LINC02086*, and *LINC01164* are novel direct p53 targets (Fig. 2A–C). Notably, some established p53‐targeted lncRNAs have not been confirmed by our study, such as *LINC‐PINT* for which a p53‐binding signal was observed further upstream, underscoring that the thresholds we employed are rather conservative.

**Table 1:** Potential direct p53‐targeted lncRNAs. Eighty‐six lncRNAs recurrently up‐regulated by p53 (p53 Expression Score ≥ 10) with a recurrent p53‐binding site within 2.5 kb from their TSS.

*AC004264.1*	*AC092171.5*	*CCDC18‐AS1*	*LINC01629*	*PCBP1‐AS1*
*AC005264.1*	*AC103760.1*	*DINOL*	*LINC01707*	*PINCR*
*AC005865.2*	*AC104134.1*	*DOCK8‐AS1*	*LINC01778*	*PRECSIT*
*AC006262.1*	*AC104304.3*	*DUBR*	*LINC02051*	*PURPL*
*AC007342.4*	*AC107959.2*	*EPB41L4A‐AS1*	*LINC02086*	*PVT1*
*AC007448.4*	*AC122719.3*	*ERVK13‐1*	*LINC02303*	*SRRM2‐AS1*
*AC008972.1*	*AC254633.1*	*FAM198B‐AS1*	*LINC02541*	*TCERG1L‐AS1*
*AC008972.2*	*AL096870.12*	*FLG‐AS1*	*LINC02846*	*TP53TG1*
*AC010271.2*	*AL109976.1*	*FLJ16779*	*LINC02875*	*TRHDE‐AS1*
*AC010624.1*	*AL132780.2*	*GARS1‐DT*	*LNCTAM34A*	*TRINGS*
*AC010624.3*	*AL135905.1*	*INKA2‐AS1*	*MIR222HG*	*TYMSOS*
*AC046134.2*	*AL157388.1*	*KLRK1‐AS1*	*MIR34AHG*	*UNC5B‐AS1*
*AC068057.1*	*AL157400.2*	*LINC00513*	*MIR3659HG*	*Z92544.1*
*AC073114.1*	*AL353138.1*	*LINC00589*	*NALT1*	*ZNF561‐AS1*
*AC074194.1*	*AL353622.1*	*LINC00663*	*NEAT1*	
*AC084082.1*	*AL450043.1*	*LINC01136*	*NINJ2‐AS1*	
*AC087752.3*	*AL731702.1*	*LINC01164*	*NOP14‐AS1*	
*AC092171.3*	*BDNF‐AS*	*LINC01173*	*PCAT1*	

#### 3.2.2 Identification of 23 lncRNAs potentially regulated through p53‐bound enhancers

In addition to binding near a gene's TSS, p53 has been shown to bind and regulate enhancers interacting with proximal gene promoters to induce gene expression [3], including genes encoding lncRNAs [60]. To identify lncRNAs potentially regulated by p53 through enhancers, we resorted to the double‐elite enhancers and enhancer:gene associations listed in the GeneHancer database [49]. We identified 23 lncRNAs consistently up‐regulated by p53 that are associated with an enhancer harboring a recurrent p53‐binding site and are located more than 2.5 kb away from the gene's TSS (Table 2). These 23 lncRNAs include *CALML3‐AS1* and *GAS6‐AS1*, which may be regulated by p53 binding to an associated enhancer that is located upstream (Fig. 2D) or intragenic (Fig. 2E), respectively.

**Table 2:** LncRNAs potentially regulated by p53 through enhancers. Twenty‐three lncRNAs recurrently up‐regulated by p53 (p53 Expression Score ≥ 10) with a recurrent p53‐binding site > 2.5 kb from their TSS that overlaps an associated enhancer.

*AC005821.1*	*AL355916.1*	*CALML3‐AS1*	*LNCAROD*	*UBR5‐AS1*
*AC012313.1*	*AL590560.3*	*GAS6‐AS1*	*LUARIS*	*WAKMAR2*
*AC023906.2*	*AP002026.1*	*LINC00649*	*MAP4K3‐DT*	*ZBTB11‐AS1*
*AC074117.1*	*ASB16‐AS1*	*LINC01588*	*PICART1*	
*AL157392.3*	*BAALC‐AS1*	*LINC02615*	*TMEM9B‐AS1*	

#### 3.2.3 LncRNAs indirectly up‐regulated through the p53‐RFX7 signaling axis

In addition to direct gene regulation, p53 employs indirect means of transcriptional regulation. Recently, a p53‐RFX7 signaling axis has been discovered, showing that p53 can mediate gene up‐regulation through the transcription factor RFX7 [9]. Target genes of RFX7 include lncRNAs such *NRSN2‐AS1* [9]. The transcript in antisense to *NRSN2* is among the 313 lncRNAs recurrently up‐regulated by p53 (Table S1). Induction of p53, *e.g.*, through Nutlin‐3a treatment, led to increased occupancy of RFX7 near a TSS of *NRSN2‐AS1* (Fig. 2F) and ultimately to an RFX7‐dependent induction of *NRSN2‐AS1* levels (Fig. 2G). In addition to *NRSN2‐AS1*, lncRNAs recurrently up‐regulated by p53 include the RFX7 targets *MIR22HG*, *PDCD4‐AS1*, and *TOB1‐AS1* [61] (Table S1).

#### 3.2.4 Identification of 17 lncRNAs potentially regulated through p53‐p21‐DREAM/RB signaling

**Fig. 3:**
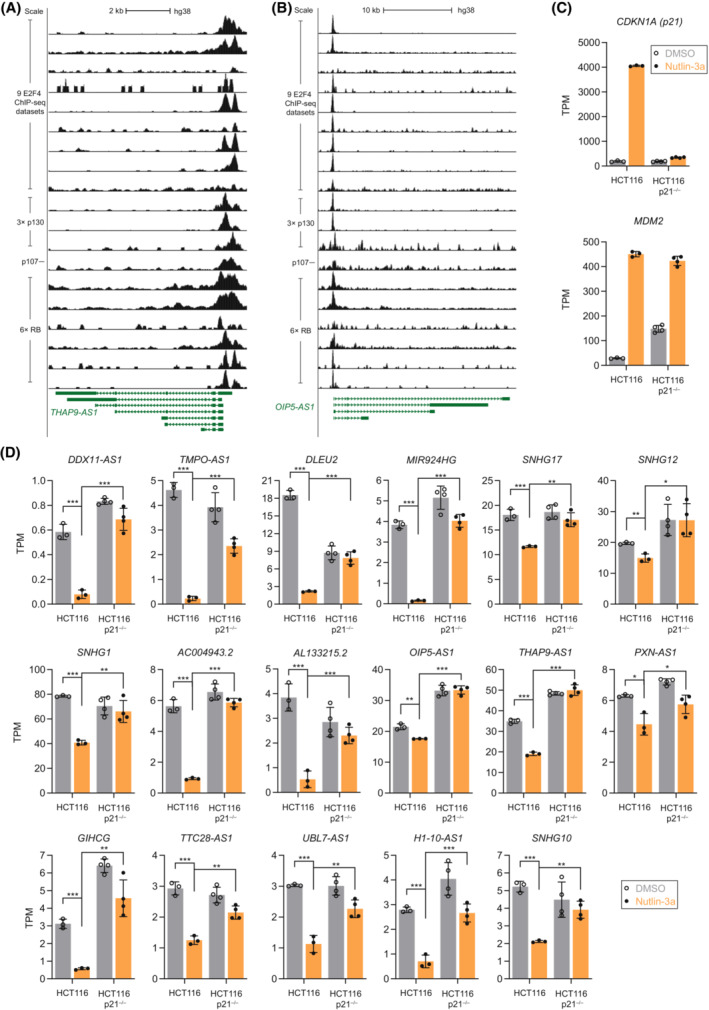
LncRNA regulation through p53‐p21‐DREAM/RB signaling. Data on E2F4, p130/p107, and RB binding from ChIP‐seq datasets [45] at the genomic loci of the recurrently down‐regulated lncRNAs (A) *THAP9‐AS1* and (B) *OIP5‐AS1*. Transcripts per million (TPM) values obtained from RNA‐seq data of Nutlin‐3a (orange) and DMSO control‐treated (gray) cells for (C) *CDKN1A (p21)* and *MDM2* and (D) 17 lncRNAs recurrently down‐regulated by p53 and bound by DREAM/RB. (C, D) RNA‐seq data from parental HCT116 [9] and HCT116 p21^−/−^ cells [8]. Bar graphs display mean and standard deviation. TPM values from RNA‐seq datasets were compared using a two‐sided unpaired Student's *t*‐test. *, **, ***, and n.s. indicate *P* values < 0.05, < 0.01, < 0.001, and > 0.05, respectively.

Many protein‐coding genes have been shown to be indirectly down‐regulated by p53 through the CDK inhibitor p21 and the *trans*‐repressor complexes DREAM (DP, RB‐like, E2F4, and MuvB) and RB:E2F [1, 3, 4, 7, 8]. It appears likely that the p53‐p21‐DREAM/RB:E2F signaling axis is not limited to protein‐coding genes. To identify lncRNAs that are potentially down‐regulated through the p53‐p21‐DREAM pathway, we integrated ChIP‐seq data on the transcriptional regulators E2F4 and p130/p107, two key repressive components of the DREAM complex [62, 63] (see Section 2). For 21 out of the 66 lncRNAs recurrently down‐regulated by p53, we observed recurrent binding signals of E2F4 and p130/p107, indicating a direct regulation of these lncRNAs through the DREAM complex. In addition, we integrated RB ChIP‐seq data and analogously identified 14 lncRNAs with recurrent RB binding near their TSS. Notably, 12 of them overlap with the set of lncRNAs identified as potential targets of E2F4 and p130/p107 (Table S1), like *THAP9‐AS1* (Fig. 3A) and *OIP5‐AS1* (Fig. 3B). To validate the joined set of lncRNAs regulated either exclusively through p53‐p21‐DREAM (*n* = 21), RB (*n* = 14) or both (*n* = 12) signaling (*n* = 23), we assessed their expression values in parental and p21 knock‐out HCT116 cells following Nutlin‐3a treatment. The direct p53 target *MDM2* is induced in both parental and p21^−/−^ HCT116 cells, while *CDKN1A* (*p21*) is properly induced only in the parental HCT116 cells (Fig. 3C). Of note, p53 levels are known to be elevated in HCT116 p21^−/−^ cells in the control condition [64], which leads to increased expression of p53 targets, such as *MDM2* (Fig. 3C). Out of the 23 DREAM/RB‐bound lncRNAs recurrently down‐regulated by p53, we found 17 to be repressed upon Nutlin‐3a treatment in parental cells. Importantly, in HCT116 p21^−/−^ cells these 17 lncRNAs displayed a substantial failure of repression compared with parental HCT116 under Nutlin‐3a treatment (Fig. 3D), indicating that those 17 lncRNAs are substantially regulated by the p53‐p21 axis. The remaining six lncRNAs showed either no down‐regulation in the parental HCT116 cells or no significant changes in repression in the HCT116 p21^−/−^ cells under Nutlin‐3a treatment (Fig. S2A).

**Fig. 4:**
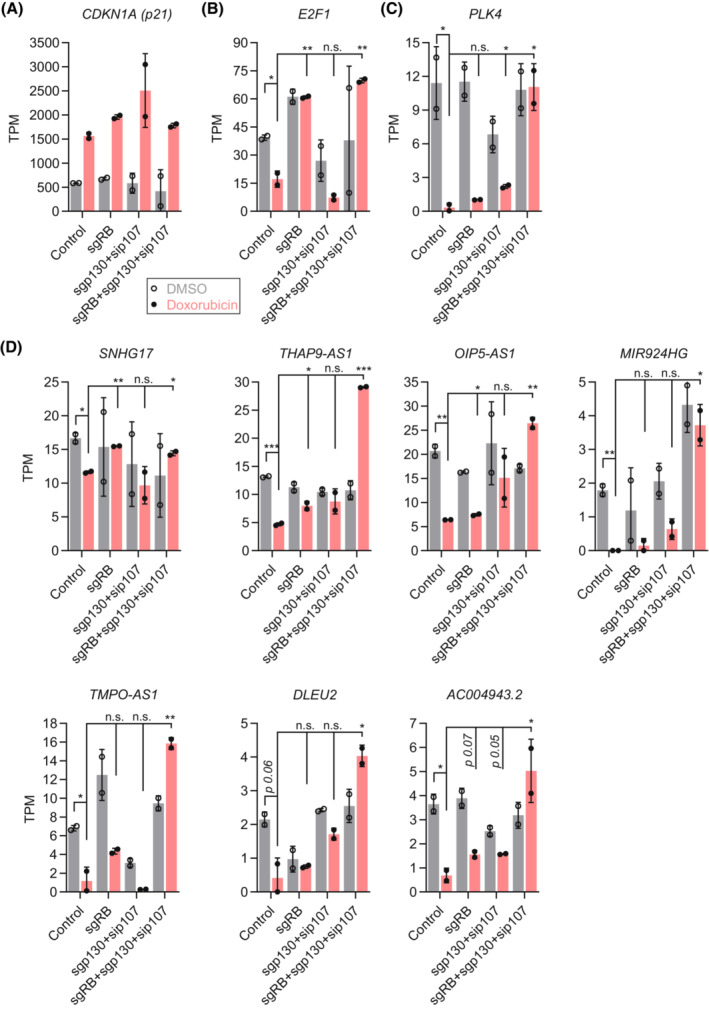
LncRNA regulation through RB and p130/p107. (A–D) Transcripts per million (TPM) values obtained from RNA‐seq data of Doxorubicin (red) and control‐treated (gray) HFF cells in which RB, p130/p107, or RB/p130/p107 were depleted. (A) *CDKN1A (p21)*, (B) *E2F1*, (C) *PLK4*, and (D) seven lncRNAs recurrently down‐regulated by p53 through p21 and bound by DREAM/RB. (A–D) Bar graphs display mean and standard deviation. TPM values from RNA‐seq datasets were compared using a two‐sided unpaired Student's *t*‐test. *, **, ***, and n.s. indicate *P* values < 0.05, < 0.01, < 0.001, and > 0.05, respectively.

To discern the role of DREAM and RB in controlling the 17 p53‐p21‐regulated lncRNAs, we resorted to Doxorubicin‐treated primary HFF in which RB, p130, and p107 were depleted [8]. The HFF cells displayed an up‐regulation of CDKN1A (p21) regardless of the availability of RB, p130, and p107 (Fig. 4A). Although DREAM and RB have redundant roles and cooperate in the p53‐dependent regulation of target genes, it has been established that RB has a predominant role in regulating G1/S cell cycle genes, while p130/p107 (key repressive components of the DREAM complex) have a more important role in regulating G2/M cell cycle genes in response to p53 activation [7, 8]. *E2F1* (Fig. 4B) [4, 8] and *PLK4* (Fig. 4C) [65] are established examples of G1/S and G2/M cell cycle genes, respectively. Out of the 17 p53‐p21‐regulated lncRNAs, we found seven to be detected and to be down‐regulated upon Doxorubicin treatment in the parental HFF cells. Loss of RB and p130/p107 appeared to have varying impact on the Doxorubicin‐mediated lncRNA repression, but the loss of all three pocket proteins abrogated the repression of all seven lncRNAs (Fig. 4D). Similar to G1/S cell cycle genes, such as *E2F1* (Fig. 4B), RB appeared to have a predominant role in regulating *SNHG17* and *TMPO‐AS1* (Fig. 4D). By contrast, p130/p107 appeared to have a more important role in regulating *OIP5‐AS1* and *MIR924HG* (Fig. 4D), which is similar to the regulation of G2/M cell cycle genes, such as *PLK4* (Fig. 4C). For the other three lncRNAs, a predominant role of either RB or DREAM could not be identified, but in most cases, RB and p130/p107 cooperated to mediate repression of the lncRNAs in response to Doxorubicin treatment (Fig. 4D). Together, the data provide evidence that multiple lncRNAs are regulated by p53 through p21, DREAM (p130/p107), and RB.

#### 3.2.5 LncRNAs potentially regulated by p53 through host genes

**Fig. 5:**
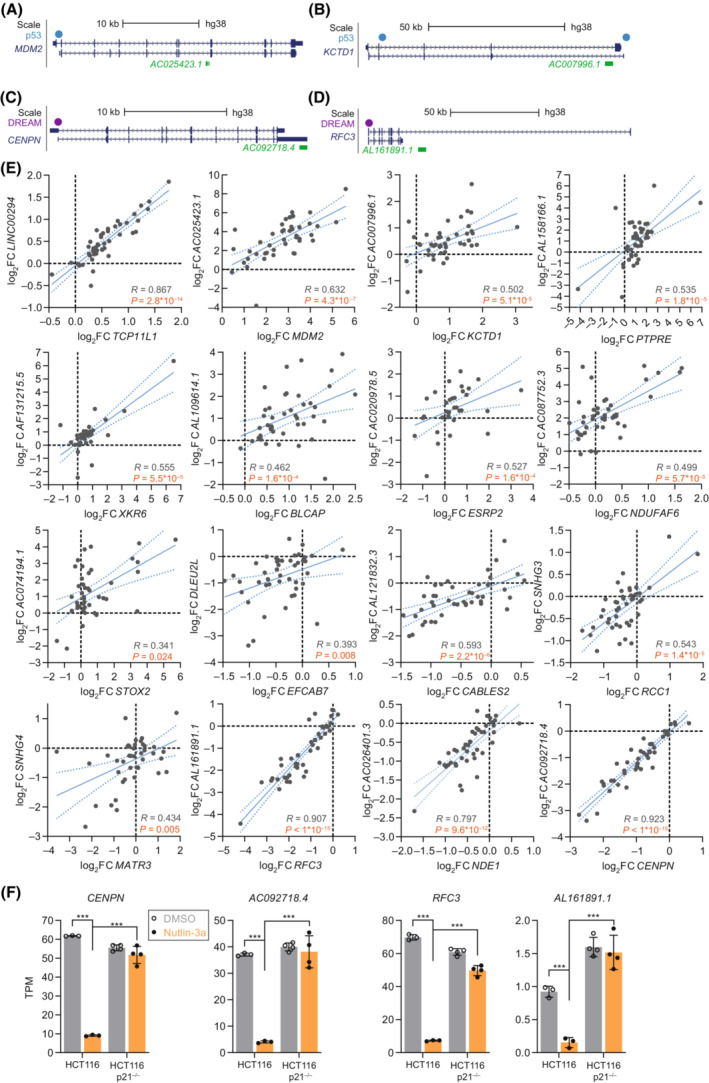
Nested lncRNAs potentially regulated by p53 through their host genes. Genomic loci of (A) *AC025423.1* hosted by the p53 target *MDM2*, (B) *AC007996.1* hosted by the p53 target *KCTD1*, (C) *AC092718.4* hosted by the DREAM target *CENPN*, and (D) *AL161891.1* hosted by the DREAM target *RFC3*. (A–D) Two representative host gene transcripts are displayed in dark blue. LncRNAs are displayed in green. Blue and purple dots indicate p53 [43, 45] and DREAM‐binding sites [4, 45], respectively. (E) Expression changes of lncRNAs (*y*‐axis) and their host genes (*x*‐axis) inferred from 44 RNA‐seq datasets for which log_2_fold‐change (log_2_FC) data was available for both genes. The blue lines indicate linear regression (solid line) and its 95% confidence interval (dotted line). The Spearman correlation (*R*) is displayed with the respective *P* value. Orange *P* values indicate 16 lncRNA/host gene pairs with significant positive expression correlation. The four lncRNA/host gene pairs that do not show significant positive correlation are displayed in Fig. S3. (F) Transcripts per million (TPM) values obtained from RNA‐seq data of Nutlin‐3a (orange) and DMSO control‐treated (gray) HCT116 cells. Bar graphs display mean and standard deviation. TPM values from RNA‐seq datasets were compared using a two‐sided unpaired Student's *t*‐test. *** indicates *P* values < 0.001.

To identify additional mechanisms by which p53 may regulate lncRNA expression, we inspected the genomic loci of recurrently up and down‐regulated lncRNAs that were no potential direct targets of p53, RFX7, or DREAM/RB. We found that multiple p53‐regulated lncRNAs were located intragenic, *i.e.*, within a host gene on the same strand, of protein‐coding genes. In general, many ncRNAs have been identified to be nested in protein‐coding genes and some are co‐regulated with their host gene [66]. Our computational analysis indicated that 12 and 8 out of the 313 and 66 recurrently up and down‐regulated lncRNAs, respectively, were nested in a protein‐coding host gene. Examples include *AC025423.1* hosted by the p53 target *MDM2* (Fig. 5A), *AC007996.1* hosted by the p53 target *KCTD1* (Fig. 5B), *AC092718.4* hosted by the DREAM target *CENPN* (Fig. 5C), and *AL161891.1* hosted by the DREAM target *RFC3* (Fig. 5D). *MDM2*, *KCTD1*, and *RFC3* host the lncRNAs in intronic regions, while *CENPN* hosts *AC092718.4* in its 3′UTR. To identify whether the 20 lncRNAs were co‐regulated with the respective host genes, we assessed the correlation of their expression values. The p53‐dependent regulation of 16 out of the 20 lncRNAs displayed a significantly positive correlation with their host genes (Fig. 5E and Fig. S3), suggesting that these lncRNAs are regulated by p53 through regulation of their respective host gene.

To further test whether nested lncRNAs are indeed co‐regulated with their host genes by common regulatory pathways, we analyzed the expression of the lncRNA/host gene pairs *AC092718.4*/*CENPN* and *AL161891.1*/*RFC3* in greater detail. Given that *CENPN* and *RFC3* have been predicted to be regulated by the p53‐p21‐DREAM signaling pathway [4], the p53‐dependent down‐regulation of both, the host genes and the nested lncRNAs, is expected to depend on the availability of p21. To this end, we again resorted to RNA expression in parental HCT116 and HCT116 p21^*−/−*^ cells upon treatment with Nutlin‐3a. The expression of both lncRNA/host gene pairs was down‐regulated in parental HCT116 cells treated with Nutlin‐3a, and displayed a failure of repression in Nutlin‐3a‐treated HCT116 p21^*−/−*^ cells (Fig. 5F), providing evidence that *AC092718.4*/*CENPN* and *AL161891.1*/*RFC3* are indeed co‐regulated by p53‐p21 signaling. Thus, regulation of lncRNA expression through a co‐regulation with their host genes appears to be an important regulatory means employed by p53.

### 3.3 Potential function and clinical relevance of lncRNAs recurrently regulated by p53

**Fig. 6:**
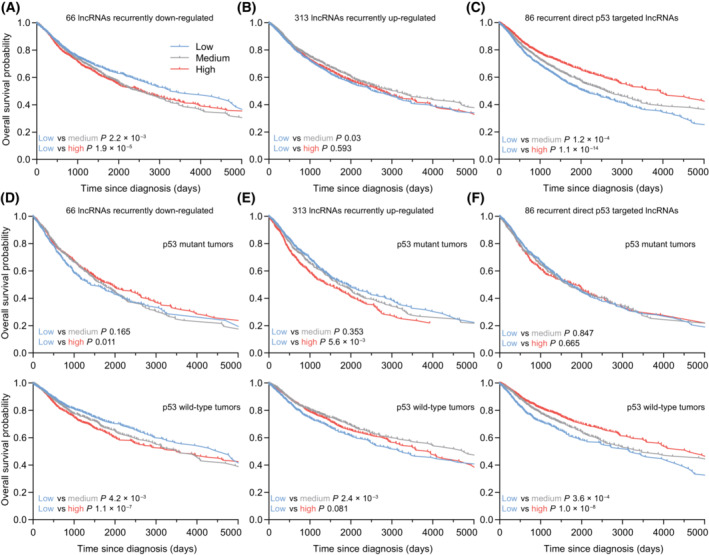
Potential function and clinical relevance of recurrently p53‐regulated lncRNAs. Kaplan–Meier plot displaying the overall survival of patients from the TCGA pan‐cancer cohort, shown until day 5000 following diagnosis. Patients were grouped into low, medium, and high based on the rank expression of (A) the 66 lncRNAs recurrently down‐regulated by p53, (B) the 313 lncRNAs recurrently up‐regulated by p53, and (C) the 86 lncRNAs identified as recurrent direct p53 targets. Patients were grouped based on their tumor's p53 status and sub‐grouped into low, medium, and high based on the rank expression of (D) the 66 lncRNAs recurrently down‐regulated by p53, (E) the 313 lncRNAs recurrently up‐regulated by p53, and (F) the 86 lncRNAs identified as recurrent direct p53 targets. (A–F) Statistical significance of the rates of occurrence of events over time between the groups was obtained using the fitted Cox PH model (Cox likelihood ratio test groups). To correct for major confounding factors, sex and age were included into the multivariate regression analysis.

Recurrent regulation by p53 may indicate the regulator's role in amplifying tumor suppressive and oncogenic roles. Thus, we hypothesized that lncRNAs recurrently up‐regulated by p53 may be more likely to promote a tumor suppressive role, while lncRNAs recurrently down‐regulated by p53 might have oncogenic potential. To test this hypothesis, we resorted to patient data from TCGA. We used the expression of recurrently up‐ and down‐regulated lncRNAs to stratify cancer patients into three groups displaying a low, medium, or high expression of the lncRNA gene sets. First, we took a look at the 66 lncRNAs recurrently down‐regulated by p53. The patients with a high or medium expression of lncRNAs typically down‐regulated by p53 displayed a significantly worse prognosis when compared with the low expression group (Fig. 6A). By contrast, a high expression of the 313 lncRNAs recurrently up‐regulated by p53, tended to correlate with a better patient prognosis (Fig. 6B). However, the group with a medium expression of this set of lncRNAs showed the best survival. Investigating the impact of the 86 lncRNAs that we identified as recurrent direct targets of p53, higher expression correlated significantly with a better prognosis. This set of lncRNAs allowed for a strong stratification of the patients (Fig. 6C). Given that these sets of lncRNAs are regulated by p53, we separated the patients according to their tumor's p53 mutation status. Intriguingly, the expression of the lncRNA sets hardly correlated with prognosis in patients with p53 mutant tumors. By contrast, patients with p53 wild‐type tumors displayed a strong negative correlation with the set of p53 down‐regulated lncRNAs (Fig. 6D) and a strong positive correlation with the sets of p53 up‐regulated lncRNAs (Fig. 6E,F). Together, these data suggest that sets of lncRNAs recurrently up and down‐regulated by p53 may be enriched for lncRNAs having a role in suppressing and promoting cancer development, respectively. Alternatively, the lncRNA expression could indicate p53 activity and only indirectly relate to p53's tumor suppressive actions.

A recent meta‐analysis of associations between lncRNAs and co‐essential modules identified 123 and 252 lncRNAs as potential inducers and suppressors of cell proliferation across cancer cell lines, respectively [67]. Our set of 66 lncRNAs recurrently down‐regulated by p53 contains 19 lncRNAs that have been identified as potential inducers of proliferation (Fisher's exact test *P* < 10^−15^), and our set of 313 lncRNAs recurrently up‐regulated by p53 contains 44 lncRNAs that have been identified as potential suppressors of proliferation (*P* < 10^−15^). These substantial overlaps provide further evidence that lncRNAs recurrently up and down‐regulated by p53 may well promote or suppress cell proliferation, respectively.

## 4 Discussion

Studies of gene regulation by p53 have largely focused on protein‐coding genes [1, 2, 3]. In the past, the study of lncRNAs has been hampered due to the lack of data and annotations caused by low‐resolution measurement platforms. In recent years, the increasing availability of high‐resolution transcriptome data led to more robust lncRNAs annotations [10, 11, 12] and facilitated studies on lncRNAs regulated by p53 [31, 32, 33, 34, 35, 36]. Since many lncRNAs are highly tissue‐specific [10, 37, 38] and poorly conserved across species [37, 38, 42], we employed a meta‐analysis strategy [4, 44] to synthesize 44 RNA‐seq datasets derived from diverse human cell lines in response to p53 activation. Thereby, we identified 379 lncRNAs that are recurrently regulated by p53. Importantly, we integrated transcription factor‐binding data to decipher the gene regulatory mechanisms by which p53 might regulate these lncRNAs. To this end, our study provides a resource of p53‐regulated lncRNAs and the mechanisms that likely underlie their regulation.

Taking gene regulatory mechanisms into account, our integrative analysis confirmed known lncRNAs as direct p53 targets (Fig. 1B) and revealed novel lncRNAs as potential direct p53 targets (Table 1), including *LINC01629*, *LINC02086*, and *LINC01164* (Fig. 2A–C). Moreover, we identified lncRNAs that appear to be regulated through p53 binding to an associated enhancer (Fig. 2D,E), a regulatory connection that is easily missed when searching lncRNAs directly targeted by p53. Regulation of the lncRNA *NRSN2‐AS1* through the p53‐RFX7 signaling axis (Fig. 2F,G) highlights that this recently discovered mechanism [9] has a broad role in mediating p53‐dependent gene regulation that extends to lncRNA regulation. Although the p53‐p21‐DREAM/RB pathway was likely to have a role in the down‐regulation of lncRNAs, to our knowledge, no target lncRNA has been identified so far. Here, we identified 17 lncRNAs that appear to be recurrently down‐regulated through p53‐p21 signaling and bound by DREAM and RB (Fig. 3D). Importantly, out of these 17 lncRNAs, those with a detectable repression signal in Doxorubicin‐treated HFF cells required the transcriptional regulators RB or the key repressive DREAM components p130/p107 to be down‐regulated by p53 (Fig. 4D). Thus, it appears likely that these lncRNAs are regulated through p53‐p21‐DREAM/RB signaling, underscoring the importance of this pathway for p53‐induced lncRNA repression. In addition to transcription factors mediating p53‐dependent regulation of lncRNAs, the lncRNAs themselves can influence gene regulation, *e.g.*, in *cis*, by affecting the chromatin state at their locus [68].

Moreover, our analysis of p53‐dependent lncRNAs suggests a host gene‐associated means of regulation. We identified 16 lncRNAs to be nested in host genes and indirectly regulated by p53 through a regulation of their host genes (Fig. 5). Notably, there are multiple mechanisms that might facilitate the co‐regulation. Nested ncRNAs can be either co‐transcribed with their host genes or rely on the same transcription initiation site as the host gene. Alternatively, a lncRNA might be independently transcribed via individual transcription initiation sites [66]. While co‐regulation of co‐transcribed nested genes is largely driven through the regulation of a shared promoter, co‐regulation of independently transcribed nested genes can occur through an enhancer that regulates both transcription initiation sites or through changes of the chromatin structure at the host gene locus. Given that short ncRNAs are found more often to be nested in host genes compared with lncRNAs [66], it is likely that p53 regulates an even larger number of ncRNAs by controlling their host genes.

An analysis of clinical cancer patient data revealed that lncRNAs recurrently regulated by p53 are associated with prognosis in patients with p53 wild‐type tumors (Fig. 6). With respect to the overall prognostic potential of lncRNAs, however, it appears to be necessary to take individual tumor types into account since lncRNA expression exhibits a strong tissue specificity [10, 37, 38]. In agreement with the tumor suppressive function of p53, lncRNAs recurrently up and down‐regulated by p53 are associated with better and worse survival probability, respectively. Intriguingly, lncRNAs directly controlled by p53 displayed a stronger association with cancer patient survival compared with all recurrently p53 up‐regulated lncRNAs. Whether this association is a direct consequence of the lncRNAs' expression or rather reflects the global activity of p53 is the subject of further research, *e.g.*, by experimentally targeting the lncRNA expression.

While lncRNAs directly regulated by p53 include multiple lncRNAs with an established tumor suppressive role (Table 1), lncRNAs recurrently down‐regulated by p53 via p21 also include host genes of small nucleolar RNA families (snoRNAs), such as *SNHG1*, *SNHG10*, *SNHG12*, and *SNHG17* (Fig. 3D). In recent years, snoRNA host genes have been associated with cancer development and progression [69, 70]. In addition to snoRNA host genes, multiple lncRNAs down‐regulated through p53‐p21 signaling have been shown to have oncogenic, pro‐proliferative functions, including *DDX11‐AS1* (*SCAT4*) [71, 72], *TMPO‐AS1* [73], *DLEU2* [74], *MIR924HG* (*LINC00669*) [75], *OIP5‐AS1* [76], *THAP9‐AS1* [77], *PXN‐AS1* [78], *GIHCG* [79], and *UBL7‐AS1* [80]. Together, our data provide evidence that recurrently p53‐regulated lncRNAs enrich tumor suppressive and oncogenic functions. Thus, they could be critical components amplifying p53's tumor suppressive activity.

## 5 Conclusions

Taken together, we identified 379 lncRNAs that are recurrently differentially regulated by p53 through a meta‐analysis of 44 RNA‐seq datasets. Integration of transcription factor‐binding data enabled us to dissect the mechanisms by which p53 regulates many of those lncRNAs. This includes sets of lncRNAs regulated directly by p53 and indirectly through the p53‐RFX7 and p53‐p21‐DREAM/RB:E2F pathways. The identification of lncRNAs that are co‐regulated with their protein‐coding host genes by p53 revealed an important novel mechanism by which p53 may regulate lncRNAs. Our resource of p53‐regulated lncRNAs contains promising candidates for functional studies and for the use as biomarkers.

## Conflict of interest

The authors declare no conflict of interest.

## Author contributions

MF conceived the study. MF and SH supervised the work and interpreted the data. MF and KR analyzed the data. MF, with the help of SH and KR, wrote the manuscript.

### Peer Review

The peer review history for this article is available at https://publons.com/publon/10.1002/1878‐0261.13405.

## Supporting information


**Fig. S1.** LncRNAs most recurrently up‐ and down‐regulated by p53.
**Fig. S2.** LncRNAs recurrently down‐regulated by p53 and bound by DREAM/RB but not clearly regulated through p21.
**Fig. S3.** Nested lncRNAs that do not display a significant positive expression correlation with their host genes.
**Table S1.** Meta‐analysis of 44 RNA‐seq analyses of p53‐dependent gene expression from human cell lines.

## Data Availability

The integrative analysis data are available through Table S1.
