# Supplementary material for: The landscape of human p53‐regulated long non‐coding RNAs reveals critical host gene co‐regulation
Source: Mol Oncol. 2023 Mar 9;17(7):1263–79. doi: 10.1002/1878-0261.13405 (PMC10323904; doi:10.1002/1878-0261.13405)
Supplement: Supplementary file 1 — Fig. S1. LncRNAs most recurrently up‐ and down‐regulated by p53. Fig. S2. LncRNAs recurrently down‐regulated by p53 and bound by DREAM/RB but not clearly regulated through p21. Fig. S3. Nested lncRNAs that do not display a significant positive expression correlation with their host genes. Table S1. Meta‐analysis of 44 RNA‐seq analyses of p53‐dependent gene expression from human cell lines. [file MOL2-17-1263-s001.zip › Supplementary Figure1.pdf]

Supplementary Figure 1

p53 Expression Score	median log2FC	Gene Symbol
44	2.481	AL158206.1
38	0.896	PVT1
37	2.542	LNCTAM34A
37	2.066	MIR34AHG
37	2.016	AL135905.1
36	0.781	BX890604.1
34	2.028	GAS6-AS1
34	1.803	AC107959.1
34	1.200	ZNF561-AS1
32	3.223	UNC5B-AS1
32	2.844	PURPL
32	2.686	TRINGS
32	2.517	AL109976.1
31	0.417	NORAD
30	2.080	AC087752.3
29	4.601	PINCR
29	1.504	Z92544.1
28	2.554	FLG-AS1
28	2.168	LINC02846
28	1.199	UBR5-AS1
28	0.806	MIR22HG
27	2.780	AL353138.1
27	1.155	AC080112.1
27	0.905	NEAT1
27	0.431	LINC00294
26	2.048	PICART1
25	1.122	TYMSOS
24	3.245	AC025423.1
24	1.196	AC008875.3
24	0.479	NOP14-AS1
23	0.873	AC087741.1
22	2.857	AC073114.1
22	1.805	LINC02875
22	1.495	NALT1
22	1.126	TP53TG1
22	1.107	LINC02086
21	2.458	LINC01629
21	2.050	FAM198B-AS1
21	1.346	INKA2-AS1
21	0.701	AC046134.2
21	0.440	LINC00174
21	0.414	NRAV
21	0.337	FTX
20	3.778	FLJ16779
20	2.607	LINC02541
20	2.372	AC007448.4
20	1.924	AC007342.4
20	1.210	LINC00663
20	0.423	AC026471.1
20	0.400	LINC02035
20	0.245	LINC00963
-15	-0.307	CRNDE
-16	-0.247	OIP5-AS1
-16	-0.263	SNHG3
-16	-0.321	THAP9-AS1
-16	-0.611	HELLPAR
-16	-1.010	AL133215.2
-17	-0.481	LINC01515
-17	-0.278	SNHG4
-17	-0.697	AC012073.1
-17	-0.881	MIR17HG
-19	-1.397	AL035461.2
-20	-0.660	AC004943.2
-21	-1.323	AL161891.1
-23	-0.578	AC026401.3
-24	-0.369	SNHG17
-24	-0.404	SNHG1
-26	-0.681	DLEU2
-26	-1.256	MIR924HG
-30	-1.395	TMPO-AS1
-31	-0.502	FGD5-AS1
-31	-0.630	AC125807.2
-31	-1.015	AC092718.4
-35	-1.193	AC091057.1

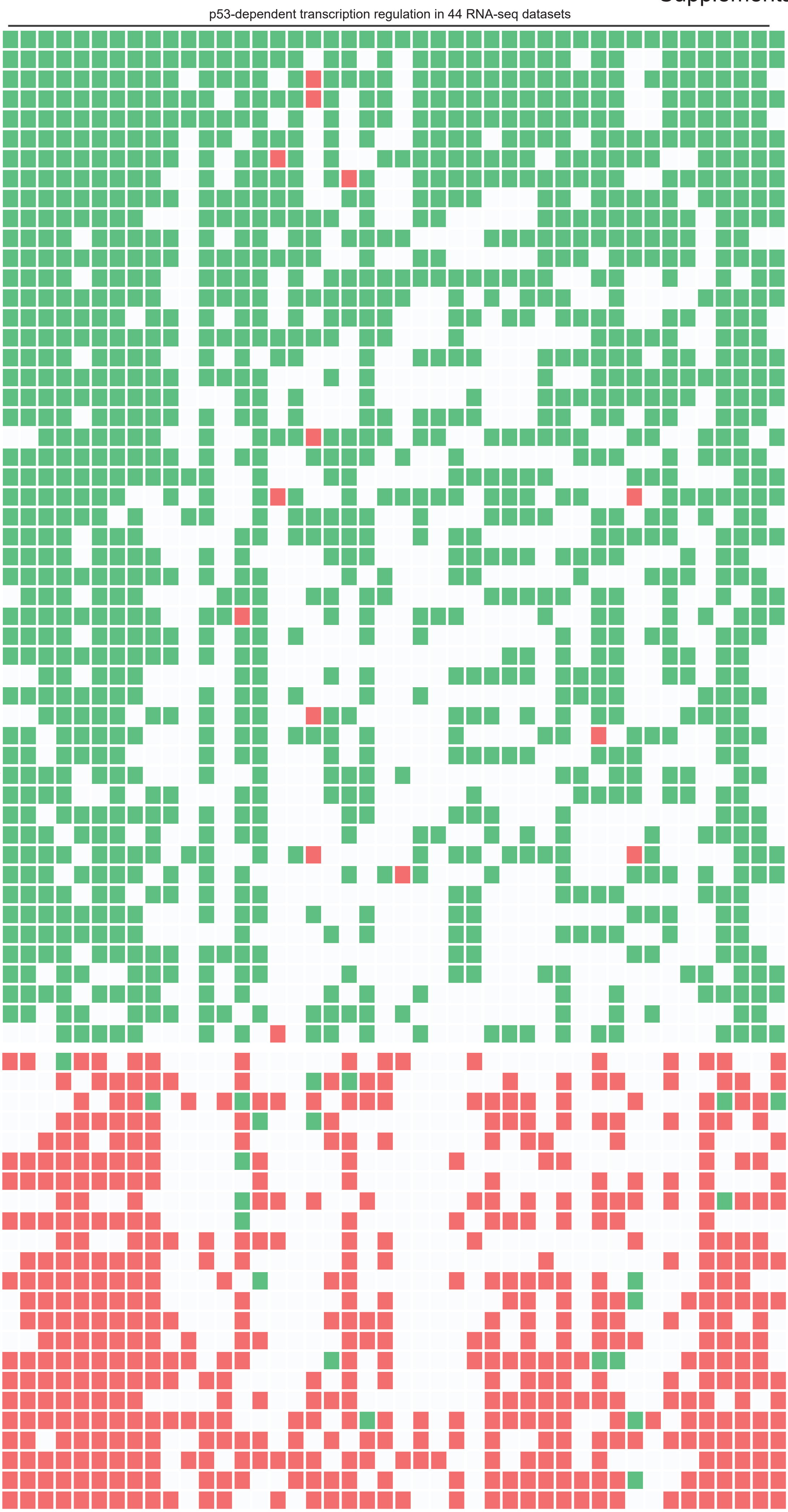

Supplementary Figure S1. LncRNAs most recurrently regulated by p53. The *p53 Expression Score* (RNA-seq) and data from the underlying 44 individual RNA-seq datasets, including the median log2fold-change (log2FC), visualized for the lncRNAs most recurrently de-regulated by p53 (*p53 Expression Score* thresholds ≥ 20 and ≤ -15).
