# Supplementary material for: The landscape of human p53‐regulated long non‐coding RNAs reveals critical host gene co‐regulation
Source: Mol Oncol. 2023 Mar 9;17(7):1263–79. doi: 10.1002/1878-0261.13405 (PMC10323904; doi:10.1002/1878-0261.13405)
Supplement: Supplementary file 1 — Fig. S1. LncRNAs most recurrently up‐ and down‐regulated by p53. Fig. S2. LncRNAs recurrently down‐regulated by p53 and bound by DREAM/RB but not clearly regulated through p21. Fig. S3. Nested lncRNAs that do not display a significant positive expression correlation with their host genes. Table S1. Meta‐analysis of 44 RNA‐seq analyses of p53‐dependent gene expression from human cell lines. [file MOL2-17-1263-s001.zip › Supplementary Figure3.pdf]

Supplementary Figure 3

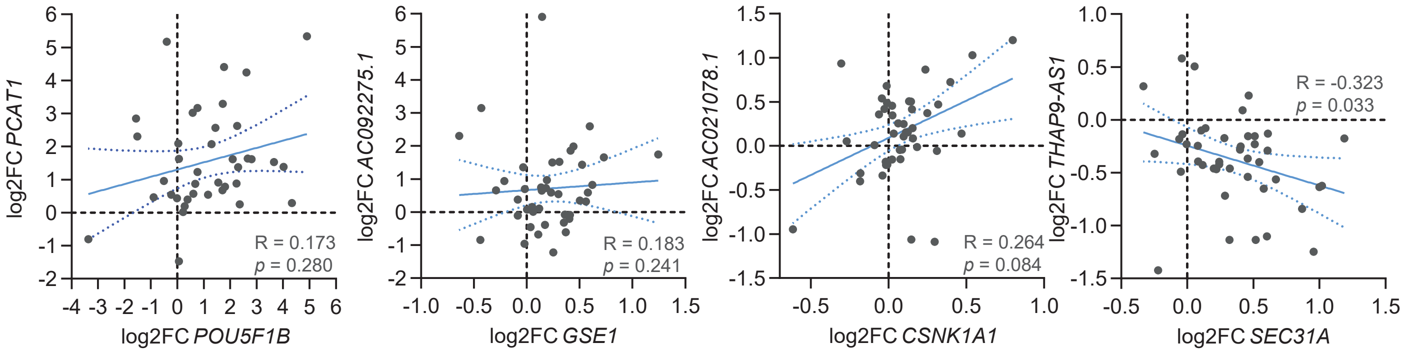

Supplementary Figure S3. Nested lncRNAs that do not display a significant positive expression correlation with their host genes. Expression changes of lncRNAs (y-axis) and their host genes (x-axis) inferred from 44 RNA-seq datasets for which log2fold-change (log2FC) data was available for both genes. The blue lines indicate linear regression (solid line) and its 95% confidence interval (dotted line). The Spearman correlation (R) is displayed with the respective *p*-value.
