# Supplementary material for: The landscape of human p53‐regulated long non‐coding RNAs reveals critical host gene co‐regulation
Source: Mol Oncol. 2023 Mar 9;17(7):1263–79. doi: 10.1002/1878-0261.13405 (PMC10323904; doi:10.1002/1878-0261.13405)
Supplement: Supplementary file 1 — Fig. S1. LncRNAs most recurrently up‐ and down‐regulated by p53. Fig. S2. LncRNAs recurrently down‐regulated by p53 and bound by DREAM/RB but not clearly regulated through p21. Fig. S3. Nested lncRNAs that do not display a significant positive expression correlation with their host genes. Table S1. Meta‐analysis of 44 RNA‐seq analyses of p53‐dependent gene expression from human cell lines. [file MOL2-17-1263-s001.zip › Supplementary Information.docx]

**Supplementary Information**

**Supplementary Table S1. Meta-analysis of 44 RNA-seq analyses of p53-dependent gene expression from human cell lines.**

**Supplementary Figure S1. LncRNAs most recurrently up and down-regulated by p53.** The *p53 Expression Score* (RNA-seq) and data from the underlying 44 individual RNA-seq datasets, including the median log2fold-change (log2FC; regardless of statistical significance), visualized for the top up and down-regulated lncRNAs. Green and red datapoints indicate significant up and down-regulation, respectively, while white datapoints indicate no significant regulation by p53.

**Supplementary Figure S2. LncRNAs recurrently down-regulated by p53 and bound by**

**DREAM/RB but not clearly regulated through p21.** Transcripts per million (TPM) values obtained from RNA-seq data of Nutlin-3a (orange) and DMSO control-treated (grey) cells for six lncRNAs recurrently down-regulated by p53 and bound by DREAM/RB.

**Supplementary Figure S3. Nested lncRNAs that do not display a significant positive expression correlation with their host genes.** Expression changes of lncRNAs (y-axis) and their host genes (x-axis) inferred from 44 RNA-seq datasets for which log2fold-change (log2FC) data was available for both genes. The blue lines indicate linear regression (solid line) and its 95% confidence interval (dotted line). The Spearman correlation (R) is displayed with the respective *p-*value.
